# Role of Polycomb Proteins in Regulating HSV-1 Latency

**DOI:** 10.3390/v5071740

**Published:** 2013-07-15

**Authors:** Zachary Watson, Adit Dhummakupt, Harald Messer, Dane Phelan, David Bloom

**Affiliations:** Department of Molecular Genetics and Microbiology, University of Florida College of Medicine, Box 100266, Gainesville, FL 32610, USA; E-Mails: levis501@ufl.edu (Z.W.); dhummaa@ufl.edu (A.D.); hmesser@ufl.edu (H.M.); dphelan@ufl.edu (D.P.)

**Keywords:** HSV, herpes, PRC, Polycomb, heterochromatin, latency, reactivation

## Abstract

Herpes simplex virus (HSV) establishes a latent infection within sensory neurons of humans. Latency is characterized by the transcriptional repression of lytic genes by the condensation of lytic gene regions into heterochromatin. Recent data suggest that facultative heterochromatin predominates, and that cellular Polycomb proteins are involved in the establishment and maintenance of transcriptional repression during latency. This review summarizes these data and discusses the implication of viral and cellular factors in regulating heterochromatin composition.

## 1. Introduction

Persistent viral infections, especially those that establish latency, utilize epigenetic modifications to regulate transcription from their latent genomes as well as to regulate their entry and exit from latency. The goals of this review are to: (1) provide an overview of epigenetic regulation of Herpes Simplex Virus type 1 (HSV-1) gene expression during latency; (2) review the evidence for polycomb proteins playing a role in regulating heterochromatin deposition on the latent genomes; and (3) discuss potential mechanisms for the remodeling of heterochromatin to facilitate selective reactivation of HSV-1 from latency.

### 1.1. Evidence for the Role of Histone Post-Translational Modifications (PTMs) in Regulating Lytic and Latent Phases of Infection

**Figure 1:**
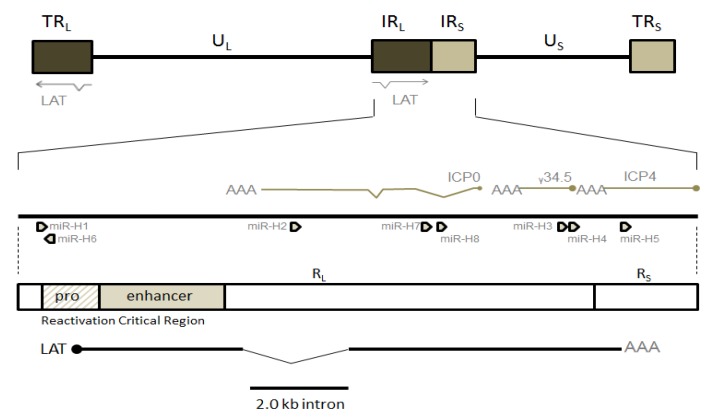
Genome and Features of HSV-1. HSV-1 is comprised of a unique long (U_L_) and unique short (U_S_) region flanked by long and short repeat regions. The primary latency-associated transcript (LAT) transcript is located in the long repeat region and is 8.5 kb long, as shown in the expanded section. Also shown are key regulatory regions for the LAT as well as the 2.0 kb LAT intron. Note that lytic gene transcripts ICP0, ICP34.5 and ICP4 and miRNAs miR-H1 through miR-H8 are present in this region.

Upon transport into the cell body of neurons, there is an ever-growing list of candidate viral and cellular factors that determine HSV’s propensity towards either productive replication or descent into latency. During the latter, the non-random distribution of histones and functionally distinct PTMs partition the roughly 152 kb HSV-1 episome into regions with different chromatin profiles. As discussed later, regions associated with the three temporal classes of lytic genes display very dense deposition of histone marks typical of facultative or constitutive heterochromatin, conveying reversible or irreversible transcriptional repression. During this time, in an estimated third of latently-infected neurons, only non-coding transcripts are transcribed from the HSV-1 genome [1,2]. These include microRNAs and long non-coding RNAs, especially the extensively studied but still equivocal latency-associated transcript (LAT) (Figure 1). The LAT is transcribed as an 8.3–8.5 kb primary transcript. A single splicing event produces a remarkably stable 2.0 kb intron which is found at high concentrations in LAT-producing neurons, and a second splicing produces a 1.5 kb intron [3,4,5]. Based on the potential interactions of the LAT with a set of counter-acting cellular and viral factors, including chromatin remodeling proteins, the LAT may mediate the pivotal balance between ultimate outcomes for the cell-virus system, including uncontrolled lytic replication (termination of the cell), irreversible episome silencing (termination of the virus), or induction of apoptosis (termination of the cell and virus). The LAT plays varying roles in the conceptual models of latency proposed by different researchers, a conflict complicated by results that don't directly compare between experimental models (discussed below).

The chromatin profile of HSV-1 at any time during infection is commonly probed using chromatin immunoprecipitation (ChIP) of desired nucleosome components followed by DNA analysis. Early applications utilized semi-quantitative methods to quantify enrichment, but qPCR using primers specific to members of different HSV-1 gene classes has become the norm. The use of high throughput sequencing (ChIP-seq) holds promise for generating HSV-1 chromatin profiles with greater resolution and coverage. Though methods of analysis for ChIP data have been converging over the last decade, there remains some controversy as to what are the most meaningful and statistically valid means of quantifying the enrichment of any one chromatin mark on the viral genome. This shortcoming complicates the assembly of data provided by independent groups into a holistic map of heterochromatin on the HSV-1 genome.

### 1.2. Animal Models Used to Study HSV-1 Latency

Despite its apparent tight co-evolution with humans, HSV-1 can produce infection in a variety of non-human animals, often with characteristic neuronal invasion and establishment of latency. For the purposes of studying HSV-1 epigenetics, mouse models are frequently utilized with the majority of data regarding latent HSV-1 chromatin dynamics generated using the footpad or corneal routes of infection. Following infection, the virus migrates along the axons of the sensory neurons afferent to these sites, and, for mice surviving the initial infection, latency is reliably and stably established in the dorsal root ganglia (DRG) or trigeminal ganglia (TG), respectively. Though the model’s consistency engenders its utility, the difficulty in detecting any clinical shedding of virus during reactivation at the primary site of infection is one of the more important dissimilarities to human pathology. Some experimentalists have developed protocols to induce reactivation via thermal stress [6], immunosuppression [7], or sodium butyrate injection [8]. Far more commonly, reactivation is induced *ex vivo*; latently infected ganglia are explanted into culture medium, where isolation from their axonal processes and natural physiological environment serves as a stressor that efficiently and synchronously induces reactivation [9,10]. Alternatively, the rabbit ocular model, though limited by its expense, is considered the most analogous to human infection, allowing for reliable clinical reactivation in live animals using ocular iontophoresis of adrenergic agents [11]. For a more thorough review of the rabbit and mouse models of latent infection, see Webre *et al*. [11].

### 1.3. *In Vitro* Models of HSV-1 Latency

Cell culture systems are also frequently employed. Cell lines used to study the HSV-1 chromatin dynamics during lytic infection include HeLa cells (human cervical carcinoma) [12,13,14,15,16], Sy5y cells (human neuroblastoma) [17,18], Vero cells (monkey kidney epithelia) [14,18] and U2OS cells (human osteosarcoma) [14]. Additionally, quiescent infection can be established *in vitro* as a means of emulating latency. For such applications, several human fibroblast cell lines have been employed, and HSV-1 can be compelled to quiescence through the use of mutant strains which lack the ability to drive expression of initial lytic genes [19,20,21] or through the provision of exogenous inhibitors of lytic replication. Addition of complementing proteins *in trans*, or removal of inhibition can, in some ways, emulate reactivation. These *in vitro* systems allow for more precise genetic assays and more defined control over the cellular and viral life cycle progression, but have acknowledged limitations on physiological relevance. More recently, the development of dissociated cultures of primary neurons either from the adult mouse TG [22] or embryonic rat superior cervical ganglia [23] show great promise in mirroring many aspects of HSV-1 latency seen *in vivo*, including the stable transcriptional repression of lytic genes and the ability to reactivate following stimulation, in a manipulable *in vitro* setting (for a review see [10]).

### 1.4. Differences in Biological Properties of Different Strains Used to Study HSV-1 Latency

Special effort is required to integrate data obtained in distinct experimental systems; cell cultures lack extrinsic factors that may influence chromatin regulation such as exogenous immune effectors and signaling molecules. The animal systems, though more holistic as models, may have developed significant differences in epigenetic regulation strategies through evolutionary divergence, and the relevance of these differences is compounded in the context of a non-equilibrium host-pathogen interaction (*i.e.*, a pathogen evolved to carefully exploit the nuances of a phylogenetically distinct host). As a notable example, an HSV-1 KOS-derived LAT mutant exhibits a 5–10 fold increase in expression of lytic genes in latently infected mouse TGs, whereas a similar mutation in a 17*syn*+-based mutant results in a 3–154 fold decrease in expression of the same genes for latently infected rabbits [24,25]. Even more subtle experimental parameters may produce distinct outcomes. For example, phenotypic differences between wild-type and LAT mutants may be observable in Swiss Webster mice but not BALB/c mice [26] or mice inoculated via the cornea but not the footpad [27]. With these issues in mind, it is essential to employ carefully considered experimental controls. In addition, sequence analysis of many of the commonly used HSV-1 strains reveal differences, some of the most dramatic of which are in the LAT region. Furthermore, as the functionality of at least some HSV-1 proteins varies among different models of infection [10] the conclusions drawn therein may have limited predictive strength with regards to human infection.

## 2. Neuronal Basis of HSV Latency

A novel feature of HSV-1, compared to other viruses that spread hematogenously, is that once the virus gains access to a nerve terminus, it enters that neuron and travels to the cell body of the neuron in the sensory ganglion. HSV-1 does not tend to spread laterally to other neurons within the ganglia and instead is limited to transynaptic spread to neurons outside the ganglion, or transport back to the epithelium. Therefore the latent reservoir of the virus is limited to those neurons in the sensory and autonomic ganglia with projections that extend to the initial site of infection at the epithelial surface; this explains why lesions tend to recur at only the initial site(s) of infection. Therefore, in the familiar orolabial route of primary infection, the latent reservoir is comprised of neurons located in the mandibular/maxillary tract of the trigeminal ganglion with axons afferent to the mouth [28]. In contrast, infections of the eye establish latency within the ophthalmic tract of the TG [29]. The molecular basis for why HSV-1 establishes latency only in neurons is not known, though it is likely due to the absence of factors in at least some neurons that promote robust activation of the lytic transcriptional program.

It is important to note that sensory neurons in the peripheral nervous system represent a very diverse and highly specialized population of cells that serve to detect a wide range of different types of sensation including hot and cold, pain, vibration, moisture, and touch. Therefore it is not surprising that there are almost 2 dozen types of sensory neurons characterized to date whose representation differs in the various sensory ganglia and individual specialized ganglia, like the TG and DRG (for a review see [30]. Also not surprisingly, HSV-1 has been shown to establish latent infection preferentially in specific populations of sensory neurons within the TG [31,32]. Studies have used antibodies specific for functional receptors such as the high-affinity nerve growth factor receptor (trkA), molecules involved in pain sensation (substance P receptor), or cell surface markers that are expressed on different sub-populations of sensory neurons to identify those neurons that are infected with HSV [32,33]. These analyses revealed that HSV-1 tends to establish latency predominately within a subclass of trkA+ neurons expressing the cell surface molecule recognized by the monoclonal antibody A5. In contrast, HSV-1 tends to initiate a predominately productive infection within neurons expressing a cell surface molecule recognized by the monoclonal antibody KH10. The molecular basis that defines these neurons as supporting productive *vs*. latent infection are not known, but point out the roles that specific cell populations play in the biology of HSV-1 latency. In addition, it is important to note that while A5+ cells represent a defined population of HSV-1 latent neurons, they make up only about 25% of the latent reservoir, and further studies are needed to define the phenotypes of the other cells which support latency, and their contribution to the pool of reactivating virus.

## 3. Chromatinization of HSV-1 Genomes during Latency

Histones are proteins that serve to package and condense DNA. The DNA strands are wrapped around an octamer core classically composed of histones H2A, H2B, H3 and H4. The large nucleosomal structure may serve to constrain or reveal binding sites depending on which histones are present and where they are placed. HSV-1 has been shown to associate with histones *in vivo*, but does not encapsulate them in the virion and thus must recruit them from the host nucleus [18]. During primary lytic infection, HSV-1 is able to associate with histones as quickly as 1 hour post infection (hpi) [18]; these histones are only loosely associated with the virus and are not spaced at regular intervals [14,34,35]. As the virus enters latency in neuronal cells, histones begin to accumulate on the viral genome in appreciable amounts at 5 days post-infection (dpi) and increase over time [36]. When treated with micrococcal nuclease, the latent viral genome produces a classic ladder pattern on a gel or Southern blot [34]. This nucleosomal pattern is similar to those of eukaryotic DNA, suggesting that histones are recruited to the virus to regulate transcription in an active and deliberate manner.

The importance that chromatin structure plays in HSV-1 latency is further evidenced by the fact that histones associated with the latent viral genome are often modified to more tightly repress genes. The histone variant macroH2A, a repressive histone subunit that replaces H2A, is enriched in latent HSV-1, especially on lytic genes [37]. Furthermore, certain amino acid residues on the N-terminus of histones can be modified, changing their interactions with proteins and nucleic acids. A common posttranslational modification (PTM) to histones that is indicative of a transcriptionally permissive state is tri-methylation of histone 3 at lysine 4 (H3K4me3). These are most enriched in the promoter of active genes [38]. Two PTMs commonly associated with gene repression are methylation of H3K9 and H3K27. H3K9me2 and H3K9me3 are abundant on constitutive heterochromatin. In eukaryotes, these are areas of the genome that are tightly condensed and largely transcriptionally silent such as the pericentromeric regions [39,40]. H3K27 methylation, on the other hand, is a hallmark of transcriptionally repressed facultative heterochromatin. These marks are more labile and are present on genes that may be activated or repressed at specific time points such as developmental genes [39]. While both of these PTMs correlate with gene repression, they are regulated by different methylases and demethylases, suggesting that the virus is using multiple pathways to silence genes.

**Figure 2:**
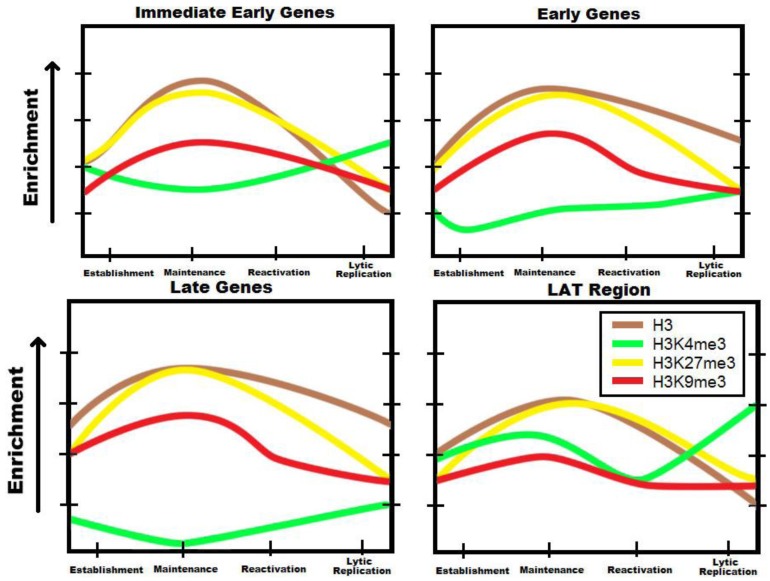
Trends in the chromatin profile of the viral genome through the HSV-1 life cycle for chosen epigenetic marks. Several groups working independently have demonstrated that the HSV-1 genome associates with a variety of investigated post-translational modifications, and that the density of any posttranslational modification (PTM) is differential with respect to each HSV-1 genetic loci and to each phase of the viral life cycle. Shown are epigenetic marks with special importance to repression during latency: histone H3, H3K4me3 (characteristic of transcriptional permissiveness), H3K27me3 (characteristic of reversible repression), and H3K9me3 (characteristic of irreversible repression). The general trends for temporal changes in enrichment of each mark are shown for the establishment of latent infection in neurons, the maintenance of transcriptional repression, the reactivation of the virus from latency, and the transition into productive replication. These trends, based on a survey of the literature for a variety of HSV-1 chromatin studies in distinct experimental models [8,12,13,14,15,16,17,18,19,20,36,37,41,42,43,44,45,46], represent a current conceptual model for regulation of latency in HSV-1. The trends for each class of transcript represent overall average enrichment for the subset of representative genes of each class examined in the cited studies. For lytic genes, deposition of histones and heterochromatic PTMs become greater during the transition to latency, but are removed as the virus reactivates. In direct contrast, investigated regions of the LAT largely exhibit increased activation and decreased repression during latency, and more modest changes in the concentration of repressive PTMs occur throughout the cycle. Note the dynamics of H3K27me3, which reaches the highest levels of enrichment and undergoes the most dramatic changes. In our conceptual model, regulation of facultative heterochromatin through deposition and removal of this mark is a central determinant of the latency/lytic replication dichotomy. It should also be noted that in many experiments, PTMs representative of all transcriptional levels have been observed converging at loci, and that this may be representative of combined input from functionally distinct populations rather than general simultaneous occurrence of the PTM on HSV-1 genomes.

ChIP has been invaluable in elucidating the abundance of PTMs on the HSV genome (Figure 2). ChIP of both H3K9 and H3K27 have both been performed by several labs. H3K9me2 has been shown to associate at around 5 dpi on the promoters of lytic genes ICP4 and TK, and substantially increases at 10 to 14 dpi through 30 dpi [36]. H3K9me3 has also been shown to be present in the LAT promoter and enhancer as well as immediate-early and early genes [37,41]. While H3K9 methylation does play a part in latency, H3K27me3 seems to play a much more prominent role. It is also present in the same genes as H3K9 methylation but in relatively higher amounts [37,41]. This PTM appears at 7 dpi; this is after initial viral association with H3 histones, suggesting that the histones are not initially methylated and are modified shortly after being recruited to the viral DNA [42]. Finally, H3K4 methylation, a mark associated with transcriptionally permissive genes, is very sparse on the genome. Interestingly, the only region found to have a relatively high abundance of H3K4me2 is in the LAT region [25]. This is logically consistent, as the LAT is abundantly transcribed during latency.

It is curious that methylation marks associated with opposing functions, and which would thus seem mutually exclusive, should occur in overlapping regions. One possible explanation is that these marks are present in a heterogeneous population of viral genomes. It is unclear whether viruses in the same cells have different chromatin marks or whether different cells harbor viruses with the same marks. This observation may also explain why the LAT region can be found with heterochromatin marks even though it is abundantly transcribed in several cells. These different PTMs may very well cause different levels of transcriptional repression. The aforementioned subpopulations of LAT-transcribing neurons might be correlated with a high enrichment of H3K4 methylation on the LAT region. Since H3K27me3 is commonly associated with facultative heterochromatin, enrichment of this mark on lytic genes may represent a loosely repressed state that allows the virus to more easily reactivate. Virus enriched in H3K9me3 may represent a smaller portion of the population that is more tightly repressed and difficult to reactivate.

## 4. Polycomb Proteins Role in Regulating Lytic Gene Activity during Latency

HSV-1 requires careful coordination of gene activity for successful lytic replication. This is also true for latency, as lytic genes must be turned off (and kept off) during latency and then turned back on in response to appropriate stressors, resulting in reactivation. The strength of lytic gene repression must be carefully modulated. If the repression is too strict, lytic genes may be silenced permanently, possibly resulting in inefficient or nonexistent reactivation and a subsequent failure to spread to further hosts. If the repression is too weak, then lytic gene activity may invite a vigorous immune response to sensory neurons harboring viral genomes (reviewed in [47]). HSV-1 has therefore adopted a strategy involving strong, yet dynamic and reversible gene silencing. During latency, the virus can utilize this strategy to escape immune surveillance. Then, during reactivation, the lytic genes can overcome the silencing for a brief burst of lytic replication to yield progeny virus.

Early studies of epigenetic regulation of HSV-1 gene expression focused primarily on DNA methylation. A broad study using methylation-sensitive restriction endonucleases found no extensive methylation of HSV-1 genomes within the central nervous systems of latently-infected mice [48]. A later study focused tightly on specific CpG dinucleotides of HSV-1 genomes within DRG of latently-infected mice [43]. No significant CpG methylation was observed. In the same study, the authors found differential histone modifications across active and inactive portions of the latent HSV-1 genome, suggesting that “histone composition may be a major regulatory determinant of HSV latency.” These studies led to the hypothesis that cellular Polycomb Group (PcG) proteins could be acting upon HSV-1 chromatin to repress lytic gene expression during latency in a manner similar to the silencing of cellular genes.

**Figure 3:**
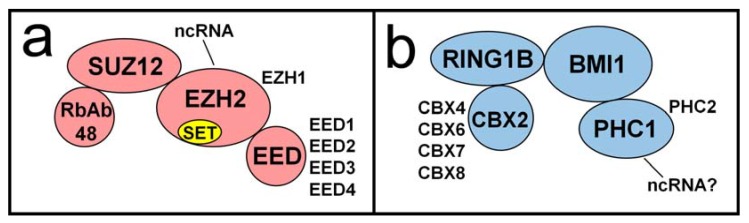
The core components of human polycomb repressive complexes are shown. Some alternate components are shown beside their more common canonical counterparts. (**a**) The SET domain of PRC2 component EZH2 catalyzes mono-, di-, and trimethylation of H3K27. EZH2 is also noted for several interactions with ncRNA; (**b**) The *C. elegans* homolog of PRC1 component PHC1 is a known RNA-binding protein. Whether or not this activity is conserved among mammalian PHC1 is under investigation.

PcG proteins, originally identified in the fruit fly *Drosophila melanogaster*, are a set of proteins that interact with and modify chromatin to effect epigenetic changes and gene silencing (extensively reviewed in [49]). PcG proteins are essential for the silencing of a multitude of cellular gene loci, including genes that determine stem cell fate, *Hox* genes and other developmental regulators, and the mammalian inactive X chromosome. They are structurally and functionally conserved throughout higher eukaryotes and primarily function through two multiprotein complexes, the Polycomb repressive complex 1 (PRC1) and Polycomb repressive complex 2 (PRC2) (Figure 3). The most widely studied complexes are those of *Drosophila* and mammals. In *Drosophila*, the complexes are somewhat simpler and consist of fewer optional or alternative subunits. They are able to recognize and bind specifically to genomic regions termed Polycomb Response Elements (PREs) [50]. In *Drosophila* these consist of 5–7 bp binding motifs for different components of the PRC2 complex including Pho/Pho1, as well as transcription factors Sp1 and KLF (for a review see [51]). Mammalian complexes are more diverse in composition and there is little evidence for binding DNA in a sequence-specific manner. Despite these differences, their activities are highly similar. PRC2 catalyzes trimethylation of lysine 27 of histone H3 (H3K27me3) [52,53]. PRC1 catalyzes ubiquitylation of lysine 119 of histone H2A (H2AK119ub) [54] and also non-enzymatically participates in compaction of polynucleosomes [55]. These histone PTMs are hallmarks of Polycomb-mediated heterochromatin and gene silencing. ChIP studies have shown H3K27me3 [37,41,42], as well as components of PRC1 [37] and PRC2 [42], to be present on latent HSV-1 genomes, strongly suggesting a role for Polycomb-based silencing of lytic genes.

The mechanisms by which histone modifications promote gene silencing are varied and still under investigation. PRC2 negatively impacts the recruitment and binding of positive chromatin regulators and the deposition of activating modifications, such as histone acetylation. A ChIP-seq analysis of PRC2-negative *Drosophila* embryos demonstrated that PRC2 blocks recruitment of RNA Pol II to promoters [56]. The lack of PRC2 and subsequent reduction in H3K27me3 resulted in the association of Pol II with promoters of over 2,000 genes that showed no association in wild-type embryos. H3K27me3 is also known to recruit PRC1 to target loci [57]. Subsequent polynucleosome compaction by PRC1 reduces accessibility of promoters and prevents transcription factor binding. However, it must be noted that this is a dynamic process and that compacted chromatin regions are not set in stone. It is a method of limiting transcriptional initiation, but it is not an absolute obstruction. This offers some explanation of “leaky” HSV-1 lytic gene transcription during latency [25], or even observations of spontaneous reactivation events [58,59].

In order to silence the correct genes, PcG proteins must first be recruited to the appropriate loci. In earlier models, PRC2 was designated the “establishment” complex where it was recruited to select loci and deposited the H3K27me3 mark. This mark then served as a binding site for PRC1, the “maintenance” complex which compacted nucleosomes and preserved silencing. While not inaccurate, later evidence shows that this model is incomplete and that PRC2 and PRC1 may be targeted by independent mechanisms [60]. In *Drosophila*, this is achieved primarily by proteins that bind specific PREs and then recruit PcG proteins. However, in mammals, only a few PRE-like elements have been identified [61,62]. It is therefore likely that most mammalian PcG association with chromatin represent more complex interactions, possibly mediated by association with specific transcription factors or other chromatin proteins. Mammalian regions associated with PcG complexes are often rich in CpG nucleotides [63] and also lack CpG methylation. As noted previously, no significant CpG methylation has been observed on latent HSV-1 genomes [43,48], perhaps opening the door for PcG recruitment. It is not yet known if HSV-1 plays an active role in preventing DNA methylation and recruiting PcG proteins to viral sequences. However, as discussed in the next section, reversible Polycomb-based gene silencing would seem to be ideal for the HSV-1 strategy of latent persistence and periodic reactivation.

PcG proteins may be targeted to select regions by multiple mechanisms, including interactions with transcription factors and non-coding RNAs (ncRNA). Several ncRNAs have been demonstrated to interact with EZH2, a member of PRC2. One example of these is RepA, a repeat region of the X-inactive specific transcript (XIST) [64]. A deletion of RepA in mice reduced H3K27me3 on the inactive X chromosome, implicating RepA in PRC2 recruitment [65]. While no direct ncRNA-PcG targeting partnership has yet been established, this is an intriguing mechanism to the field of HSV-1 epigenetics due to the high abundance and stability of the LAT RNA within sensory neurons, the site of HSV-1 latency. There is evidence that the LAT RNA, provided *in trans* in transgenic mice, reduces H3K27me3 enrichment on latent viral genomes. There is also evidence for an interaction between the stable 2.0 kb LAT intron and PRC1 component PHC1 (unpublished data). The nature of this interaction and whether it directly impacts lytic gene silencing is still under investigation.

Several mechanisms have been proposed for the observed effects of LAT transcription on PcG-based silencing of lytic genes. One study, using HSV-1 strain KOS in a mouse ocular/TG model of infection, found that a deletion of the LAT promoter resulted in decreased H3K27me3 at lytic genes [41]. It was proposed that the LAT promoted the formation of heterochromatin at lytic gene promoters, paralleling the activity of RepA in recruiting PRC2 to the inactive X chromosome. Another study, using HSV-1 strain 17*syn*+ in a mouse footpad/DRG model of infection, published seemingly contradictory results [37]. Deletion of the LAT promoter resulted in significantly higher enrichment of H3K27me3 at lytic genes, suggesting that the LAT was acting to reduce silencing of lytic genes and maintain them in a state “poised” for reactivation. In this case, the LAT may act as a decoy to misdirect Polycomb-mediated silencing. Another possibility is that the LAT RNA may associate with transcription factors or histone demethylases during latency and target them to lytic genes to increase the efficiency of reactivation. This hypothesis is consistent with induced reactivation experiments in the rabbit eye model in which 17*syn*+ reactivates efficiently but 17ΔPst, the LAT deletion mutant, does not [66]. All of these hypotheses are intriguing and are the subject of further study.

As previously noted, the above studies used strains of virus that are already known to differ greatly in virulence and reactivation potential. The fact that they behave differently in regard to PcG recruitment and heterochromatin deposition is not surprising. The differing routes of infection and sites of latency must also be taken into account. The subunit composition of Polycomb complexes is likely to differ between TG and DRG, and perhaps even between subtypes of neurons within these tissues. These cell specific differences may explain why the PRC1 component Bmi1 was found to associate with latent genomes in one study [37], but not in another [41]. On a broader scale, differential use of Polycomb subunits may explain why LAT expression is observed in only one-third of infected neurons [1,67]. It may even be a factor in the observed anatomical preference of HSV-1 for the orofacial region and HSV-2 for the genital region.

## 5. The Predicted Role of Histone Demethylases in HSV-1 Reactivation

There are currently several histone demethylases with a large assortment of names but relatively focused functions. Homologs have been identified in several organisms including *H. sapiens*, *M. musculus*, *D. melanogaster*, *C. elegans*, *S. pombe*, *S. cervisiae*, as well as some prokaryotes. Their presence in the latter suggests an ancestral role divergent from histone modification. The following section will review the relevant demethylases with potential roles in regulation of alpha-herpesvirus infections; LSD1 (lysine demethylase 1, KDM1), JMJD3 (jumonji domain containing 3, KDM6B), and UTX (ubiquitously transcribed tetratricopeptide repeat, X chromosome; KDM6A) histone demethylases. The known histone demethylases that would be predicted to play a role in remodeling HSV chromatin to facilitate reactivation are described in Table 1. 

**Table 1:** The predicted role of histone demethylases in HSV-1 reactivation.

Demethylase	Family	Specificity	Associated Complex	Biological Role	Inhibitor
LSD1 (KDM1A)	FAD-amine oxidase	H3K4me2/me1 H3K9me2/me1	HDAC1/ CoREST/ REST	Possible coordinated role with HDACs in transcription repression	Paraglyine ^a^, TCP, OG-L002
JHD3A/JMJD2A (KDM4A) JHD3C/JMJD2C/ GASC1 (KDM4C)	Jumonji ^b^	H3K9me3/me2 H3K36me3/me2 H1.4K26me ^c^	NCoR complex ^d^	KDM4C is a possible oncogene ^e^	PCA, NOG, DMOG, ML324
JARID1A/RBP2 (KDM5A) JARID1C/SMCX (KDM5C)	Jumonji	H3K4me3/me2	Sin3/HDAC complex (KDM5A) NCoR/REST (KDM5C)	Notch signaling (JARID1A) NCoR-SMCX-REST complex functions in glial development	N/A ^f^
UTX (KDM6A)	Jumonji	H3K27me3	MLL3/4, RbBP5, WDR5, and ASH2	Pluripotent stem cell differentiation (Hox gene regulation)	GSK-J4
JMJD3(KDM6B)	Jumonji	H3K27me3	RbBP5	Induced upon activation of macrophages by inflammatory stimuli Role in neuronal commitment	GSK-J4

^a^ Monoamine-oxidase inhibitor; ^b^ Dioxygenases containing a Jumonji C(JmjC) domain with an active site containing Fe(II) and the co-factor α-ketoglutaric acid; ^c^ Somatic H1 isotype in humans; ^d^ NCoR has role in neural differentiation and hematopoiesis [68]. ^e^ Gene up regulated in cell lines derived from esophageal squamous carcinomas [69]. ^f^ No publication to date.

### 5.1. FAD-Amine Oxidase: Lysine Specific Demethylase 1 (LSD1/KDM1)

Lysine-specific demethylase 1 (LSD1) specifically demethylates H3K4me2/me1 as well as H3K9me2/me1 and is unable to demethylate tri-methylated lysine due to biochemical and biophysical constraints. In addition, LSD1 is dependent on protein partners within the Set1/MLL methyltransferase complex such as the transcriptional coactivator host cell factor-1 for its activity *in vivo* and requires the neuronal silencer co-repressor of RE1-silencing transcription (CoREST) factor to demethylate nucleosome-associated histones [70]. The use of small interference RNA to reduce levels of Set1 decreases levels of H3K4me3 and ultimately reduces replication of HSV-1 [13]. LSD1 also associates with the HDAC1-2/CoREST/REST complex and suggests a role with deacetylation. Inhibition of HDACs results in concomitant decreases in LSD1-mediated demethylation [71]. Displacement of HDAC1 from the complex by the immediate-early protein, ICP0, allows for association of components of the repressor complex—either HDAC1-2/LSD1/CoREST/REST or LSD1/CoREST—with ICP8 and may play a role in the emergence of DNA-replication compartments in HSV-1 infected cells [72]. RNAi depletion of LSD1 or inhibition of its enzymatic activity by monoamine-oxidases inhibitors or the highly selective OG-L002 increases enrichment levels of repressive chromatin and blocks viral gene expression for Varicella zoster virus, HSV-1, and Cytomegalovirus [72,73]. 

### 5.2. Jumonji-Domain Histone Demethylases: JMJD3 (KDM6B) and UTX (KDM6A)

The vast majority of lysine demethylases contain a conserved Jumonji-C domain motif (JmjC). The existence of a large family of Jumonji proteins that can demethylate mono-, di-, and tri-methylated lysine in a reaction mediated by Fe(II) and α-ketoglutarate catalysis provides an additional demethylation mechanism fundamentally different from LSD1. 

UTX and JMJD3 belong to a subfamily of proteins that require a catalytically active JmjC domain to maintain demethylase activity. As well as possessing 84% sequence similarity, their Jumonji-domains share high structural conservation. Temporal expression of Hox genes—which are silenced in pluripotent cells—is mediated through the demethylation of H3K27me3 by UTX and is critical in mammalian embryogenesis. In addition to having a role in activated macrophages, JMJD3 has been identified as a protein specifically upregulated at the outset of neural commitment [74,75]. Previous studies have established that recombinant human UTX and JMJD3 that were overexpressed and purified from mammalian cells specifically remove methyl marks on H3K27 *in vitro* [76]. Decreases in di- and tri-methylation suggest that both UTX and JMJD3 may function as H3K27 demethylases *in vivo*. The association of UTX and JMJD3 with the H3K4 methyltransferase MLL family of proteins and components of the MLL complex (WDR5, RbBP5, and ASH2) suggests a physical role in balancing activation and repression [75] and may be a general phenomenon for most histone demethylases. It is unclear whether the switching of histone methyltransferase with demethylase activity on bivalent marks is cell signal specific or a result of high levels of PRC1/PRC2 within a cell—the maintenance of which is subverted by the HSV-1 LAT.

## 6. Summary and Discussion

The analysis of HSV-1 epigenomes during latency reveals a general consensus that: (1) the HSV-1 lytic genes are associated with heterochromatic histone modifications; (2) while both constitutive (H3K9me3) and facultative (H3K27me3) marks are present, the H3K27me3 marks predominate; and (3) the LAT-encoding regions of the genome display bivalent modifications of both repressive (H3K27me3) and active (H3K4me3 and H3K9,K14Ac) marks. 

A number of studies suggest that a transient and rather global re-modeling of both the lytic and latent gene regions occur rapidly following stressors that induce reactivation, though productive reactivation occurs in only a fraction (<5%) of latently infected neurons. These observations suggest that while there are initial stress-induced changes in chromatin to a large proportion of the genomes, there are downstream effectors of productive phase transcription that operate only in a sub-set of cells. Whether this reflects different neuronal population that regulate transcription differently, differences in established epigenome profiles that vary from cell to cell, or the magnitude of stress-induced signaling that reaches a given cell remains to be determined, and ultimately may require single-cell analyses to sort out. None-the-less, the identification of the spectrum of epigenetic marks that are present on the latent genomes sets the stage for identifying the remodeling proteins that ultimately play an essential role in the initial stages of HSV reactivation. 

## References

[1] Gressens P., Martin J.R. (1994). *In situ* polymerase chain reaction: Localization of HSV-2 DNA sequences in infections of the nervous system. J. Virol. Methods.

[2] Mehta A., Maggioncalda J., Bagasra O., Thikkavarapu S., Saikumari P., Valyi-Nagy T., Fraser N.W., Block T.M. (1995). *In situ* DNA PCR and RNA hybridization of herpes simplex virus sequences in trigeminal ganglia of latently infected mice. Virology.

[3] Stevens J.G., Wagner E.K., Devi R.G.B., Cook M.L., Feldman L.T. (1987). RNA complementary to a herpesvirus alpha gene mRNA is prominent in latently infected neurons. Science.

[4] Farrell M.J., Dobson A.T., Feldman L.T. (1991). Herpes simplex virus latency-associated transcript is a stable intron. Proc. Natl. Acad. Sci. USA.

[5] Wechsler S.L., Nesburn A.B., Watson R., Slanina S.M., Ghiasi H. (1988). Fine mapping of the latency-related gene of herpes simplex virus type 1: Alternative splicing produces distinct latency-related RNAs containing open reading frames. J. Virol..

[6] Sawtell N.M., Thompson R.L. (1992). Rapid *in vivo* reactivation of herpes simplex virus in latently infected murine ganglionic neurons after transient hyperthermia. J. Virol..

[7] Shimeld C., Hill T.J., Blyth W.A., Easty D.L. (1990). Reactivation of latent infection and induction of recurrent herpetic eye disease in mice. J. Gen. Virol..

[8] Neumann D.M., Bhattacharjee P.S., Giordani N.V., Bloom D.C., Hill J.M. (2007). *In vivo* changes in the patterns of chromatin structure associated with the latent herpes simplex virus type 1 genome in mouse trigeminal ganglia can be detected at early times after butyrate treatment. J. Virol..

[9] Sawtell N.M., Thompson R.L. (2004). Comparison of herpes simplex virus reactivation in ganglia in vivo and in explants demonstrates quantitative and qualitative differences. J. Virol..

[10] Wilson A.C., Mohr I. (2012). A cultured affair: HSV latency and reactivation in neurons. Trends Microbiol..

[11] Webre J.M., Hill J.M., Nolan N.M., Clement C., McFerrin H.E., Bhattacharjee P.S., Hsia V., Neumann D.M., Foster T.P., Lukiw W.J. (2012). Rabbit and mouse models of HSV-1 latency, reactivation, and recurrent eye diseases. J. Biomed. Biotechnol..

[12] Herrera F.J., Triezenberg S.J. (2004). VP16-dependent association of chromatin-modifying coactivators and underrepresentation of histones at immediate-early gene promoters during herpes simplex virus infection. J. Virol..

[13] Huang J., Kent J.R., Placek B., Whelan K.A., Hollow C.M., Zeng P.Y., Fraser N.W., Berger S.L. (2006). Trimethylation of histone H3 lysine 4 by Set1 in the lytic infection of human herpes simplex virus 1. J. Virol..

[14] Cliffe A.R., Knipe D.M. (2008). Herpes simplex virus ICP0 promotes both histone removal and acetylation on viral DNA during lytic infection. J. Virol..

[15] Kutluay S.B., Triezenberg S.J. (2009). Regulation of histone deposition on the herpes simplex virus type 1 genome during lytic infection. J. Virol..

[16] Hancock M.H., Cliffe A.R., Knipe D.M., Smiley J.R. (2010). Herpes simplex virus VP16, but not ICP0, is required to reduce histone occupancy and enhance histone acetylation on viral genomes in U2OS osteosarcoma cells. J. Virol..

[17] Kent J.R., Zeng P.Y., Atanasiu D., Gardner J., Fraser N.W., Berger S.L. (2004). During lytic infection herpes simplex virus type 1 is associated with histones bearing modifications that correlate with active transcription. J. Virol..

[18] Oh J., Fraser N.W. (2008). Temporal association of the herpes simplex virus genome with histone proteins during a lytic infection. J. Virol..

[19] Coleman H.M., Connor V., Cheng Z.S., Grey F., Preston C.M., Efstathiou S. (2008). Histone modifications associated with herpes simplex virus type 1 genomes during quiescence and following ICP0-mediated de-repression. J. Gen. Virol..

[20] Ferenczy M.W., DeLuca N.A. (2011). Reversal of heterochromatic silencing of quiescent herpes simplex virus type 1 by ICP0. J. Virol..

[21] Preston C.M., Efstathiou S., Arvin A., Campadelli-Fiume G., Mocarski E., Moore P.S., Roizman B., Whitley R., Yamanishi K. (2007). Molecular Basis of HSV Latency and Reactivation. Human Herpesviruses: Biology, Therapy, and Immunoprophylaxis.

[22] Bertke A.S., Swanson S.M., Chen J., Imai Y., Kinchington P.R., Margolis T.P. (2011). A5-positive primary sensory neurons are nonpermissive for productive infection with herpes simplex virus 1 *in vitro*. J. Virol..

[23] Kobayashi M., Kim J.Y., Camarena V., Roehm P.C., Chao M.V., Wilson A.C., Mohr I. (2012). A primary neuron culture system for the study of herpes simplex virus latency and reactivation. J. Vis. Exp..

[24] Chen S.H., Kramer M.F., Schaffer P.A., Coen D.M. (1997). A viral function represses accumulation of transcripts from productive-cycle genes in mouse ganglia latently infected with herpes simplex virus. J. Virol..

[25] Giordani N.V., Neumann D.M., Kwiatkowski D.L., Bhattacharjee P.S., McAnany P.K., Hill J.M., Bloom D.C. (2008). During HSV-1 infection of rabbits, the ability to express the LAT increases latent-phase transcription of lytic genes. J. Virol..

[26] Perng G.C., Esmaili D., Slanina S.M., Yukht A., Ghiasi H., Osorio N., Mott K.R., Maguen B., Jin L., Nesburn A.B. (2001). Three herpes simplex virus type 1 latency-associated transcript mutants with distinct and asymmetric effects on virulence in mice compared with rabbits. J. Virol..

[27] Sawtell N.M., Thompson R.L. (1992). Herpes simplex virus type 1 latency associated transcription unit promotes anatomical site-dependent establishment and reactivation from latency. J. Virol..

[28] Kolokotronis A., Doumas S. (2006). Herpes simplex virus infection, with particular reference to the progression and complications of primary herpetic gingivostomatitis. Clin. Microbiol. Infect..

[29] LaVail J.H., Zhan J., Margolis T.P. (1990). HSV (type 1) infection of the trigeminal complex. Brain Res..

[30] Raible D.W., Ungos J.M. (2006). Specification of sensory neuron cell fate from the neural crest. Adv. Exp. Med. Biol..

[31] Margolis T.P., Dawson C.R., LaVail J.H. (1992). Herpes simplex viral infection of the mouse trigeminal ganglion. Immunohistochemical analysis of cell populations. Invest. Ophthalmol. Vis. Sci..

[32] Yang L., Voytek C.C., Margolis T.P. (2000). Immunohistochemical analysis of primary sensory neurons latently infected with herpes simplex virus type 1. J. Virol..

[33] Margolis T.P., Sedarati F., Dobson A.T., Feldman L.T., Stevens J.G. (1992). Pathways of viral gene expression during acute neuronal infection with HSV-1. Virology.

[34] Deshmane S.L., Fraser N.W. (1989). During latency, herpes-simplex virus Type-1 DNA is associated with nucleosomes in a chromatin structure. J. Virol..

[35] Lacasse J.J., Schang L.M. (2012). Herpes simplex virus 1 DNA is in unstable nucleosomes throughout the lytic infection cycle, and the instability of the nucleosomes is independent of DNA replication. J. Virol..

[36] Wang Q.Y., Zhou C., Johnson K.E., Colgrove R.C., Coen D.M., Knipe D.M. (2005). Herpesviral latency-associated transcript gene promotes assembly of heterochromatin on viral lytic-gene promoters in latent infection. Proc. Natl. Acad. Sci. USA.

[37] Kwiatkowski D.L., Thompson H.W., Bloom D.C. (2009). The polycomb group protein Bmi1 binds to the herpes simplex virus 1 latent genome and maintains repressive histone marks during latency. J. Virol..

[38] Barski A., Cuddapah S., Cui K., Roh T.-Y., Schones D.E., Wang Z., Wei G., Chepelev I., Zhao K. (2007). High-resolution profiling of histone methylations in the human genome. Cell.

[39] Quina A.S., Buschbeck M., Di Croce L. (2006). Chromatin structure and epigenetics. Biochem. Pharmacol..

[40] Bartova E., Krejci J., Harnicarova A., Galiova G., Kozubek S. (2008). Histone modifications and nuclear architecture: A review. J. Histochem. Cytochem..

[41] Cliffe A.R., Garber D.A., Knipe D.M. (2009). Transcription of the herpes simplex virus latency-associated transcript promotes the formation of facultative heterochromatin on lytic promoters. J. Virol..

[42] Cliffe A.R., Coen D.M., Knipe D.M. (2013). Kinetics of facultative heterochromatin and polycomb group protein association with the herpes simplex viral genome during establishment of latent infection. MBio.

[43] Kubat N.J., Tran R.K., McAnany P., Bloom D.C. (2004). Specific histone tail modification and not DNA methylation is a determinant of herpes simplex virus type 1 latent gene expression. J. Virol..

[44] Kubat N.J., Amelio A.L., Giordani N.V., Bloom D.C. (2004). The herpes simplex virus type 1 latency-associated transcript (LAT) enhancer/rcr is hyperacetylated during latency independently of LAT transcription. J. Virol..

[45] Amelio A.L., Giordani N.V., Kubat N.J., O’Neil J.E., Bloom D.C. (2006). Deacetylation of the herpes simplex virus type 1 latency-associated transcript (LAT) enhancer and a decrease in LAT abundance precede an increase in ICP0 transcriptional permissiveness at early times postexplant. J. Virol..

[46] Creech C.C., Neumann D.M. (2010). Changes to euchromatin on LAT and ICP4 following reactivation are more prevalent in an efficiently reactivating strain of HSV-1. PLoS One.

[47] St Leger A.J., Hendricks R.L. (2011). CD8+ T cells patrol HSV-1-infected trigeminal ganglia and prevent viral reactivation. J. Neurovirol..

[48] Dressler G.R., Rock D.L., Fraser N.W. (1987). Latent herpes simplex virus type 1 DNA is not extensively methylated *in vivo*. J. Gen. Virol..

[49] Simon J.A., Kingston R.E. (2013). Occupying chromatin: Polycomb mechanisms for getting to genomic targets, stopping transcriptional traffic, and staying put. Mol. Cell.

[50] Oktaba K., Gutierrez L., Gagneur J., Girardot C., Sengupta A.K., Furlong E.E., Muller J. (2008). Dynamic regulation by polycomb group protein complexes controls pattern formation and the cell cycle in Drosophila. Dev. Cell.

[51] Ringrose L., Paro R. (2007). Polycomb/Trithorax response elements and epigenetic memory of cell identity. Development.

[52] Cao D., Wang Z., Zhang C.L., Oh J., Xing W., Li S., Richardson J.A., Wang D.Z., Olson E.N. (2005). Modulation of smooth muscle gene expression by association of histone acetyltransferases and deacetylases with myocardin. Mol. Cell Biol..

[53] Czermin B., Melfi R., McCabe D., Seitz V., Imhof A., Pirrotta V. (2002). Drosophila enhancer of Zeste/ESC complexes have a histone H3 methyltransferase activity that marks chromosomal Polycomb sites. Cell.

[54] Cao R., Tsukada Y., Zhang Y. (2005). Role of Bmi-1 and Ring1A in H2A ubiquitylation and hox gene silencing. Mol. Cell.

[55] Francis N.J., Kingston R.E., Woodcock C.L. (2004). Chromatin compaction by a polycomb group protein complex. Science.

[56] Chopra V.S., Hendrix D.A., Core L.J., Tsui C., Lis J.T., Levine M. (2011). The polycomb group mutant esc leads to augmented levels of paused Pol II in the Drosophila embryo. Mol. Cell.

[57] Fischle W., Wang Y., Jacobs S.A., Kim Y., Allis C.D., Khorasanizadeh S. (2003). Molecular basis for the discrimination of repressive methyl-lysine marks in histone H3 by Polycomb and HP1 chromodomains. Genes Dev..

[58] Margolis T.P., Elfman F.L., Leib D., Pakpour N., Apakupakul K., Imai Y., Voytek C. (2007). Spontaneous reactivation of herpes simplex virus type 1 in latently infected murine sensory ganglia. J. Virol..

[59] Feldman L., Ellison A.R., Voytek C.C., Yang L., Krause P., Margolis T.P. (2002). Spontaneous molecular reactivation of herpes simplex virus type 1 latency in mice. Proc. Natl. Acad. Sci. USA.

[60] Gao Z., Zhang J., Bonasio R., Strino F., Sawai A., Parisi F., Kluger Y., Reinberg D. (2012). PCGF homologs, CBX proteins, and RYBP define functionally distinct PRC1 family complexes. Mol. Cell.

[61] Sing A., Pannell D., Karaiskakis A., Sturgeon K., Djabali M., Ellis J., Lipshitz H.D., Cordes S.P. (2009). A vertebrate Polycomb response element governs segmentation of the posterior hindbrain. Cell.

[62] Woo C.J., Kharchenko P.V., Daheron L., Park P.J., Kingston R.E. (2010). A region of the human HOXD cluster that confers polycomb-group responsiveness. Cell.

[63] Ku M., Koche R.P., Rheinbay E., Mendenhall E.M., Endoh M., Mikkelsen T.S., Presser A., Nusbaum C., Xie X., Chi A.S. (2008). Genomewide analysis of PRC1 and PRC2 occupancy identifies two classes of bivalent domains. PLoS Genet..

[64] Zhao J., Sun B.K., Erwin J.A., Song J.J., Lee J.T. (2008). Polycomb proteins targeted by a short repeat RNA to the mouse X chromosome. Science.

[65] Zhao J., Ohsumi T.K., Kung J.T., Ogawa Y., Grau D.J., Sarma K., Song J.J., Kingston R.E., Borowsky M., Lee J.T. (2010). Genome-wide identification of polycomb-associated RNAs by RIP-seq. Mol. Cell.

[66] Bloom D.C., Devi-Rao G.B., Hill J.M., Stevens J.G., Wagner E.K. (1994). Molecular analysis of herpes simplex virus type 1 during epinephrine induced reactivation of latently infected rabbits *in vivo*. J. Virol..

[67] Maggioncalda J., Mehta A., Su Y.H., Fraser N.W., Block T.M. (1996). Correlation between herpes simplex virus type 1 rate of reactivation from latent infection and the number of infected neurons in trigeminal ganglia. Virology.

[68] Hermanson O., Jepsen K., Rosenfeld M.G. (2002). N-CoR controls differentiation of neural stem cells into astrocytes. Nature.

[69] Yang Z.Q., Imoto I., Fukuda Y., Pimkhaokham A., Shimada Y., Imamura M., Sugano S., Nakamura Y., Inazawa J. (2000). Identification of a novel gene, GASC1, within an amplicon at 9p23–24 frequently detected in esophageal cancer cell lines. Cancer Res..

[70] Gu H., Roizman B. (2009). Engagement of the lysine-specific demethylase/HDAC1/CoREST/REST complex by herpes simplex virus 1. J. Virol..

[71] Shi Y.J., Matson C., Lan F., Iwase S., Baba T., Shi Y. (2005). Regulation of LSD1 histone demethylase activity by its associated factors. Mol. Cell.

[72] Liang Y., Vogel J.L., Narayanan A., Peng H., Kristie T.M. (2009). Inhibition of the histone demethylase LSD1 blocks alpha-herpesvirus lytic replication and reactivation from latency. Nat. Med..

[73] Liang Y., Quenelle D., Vogel J.L., Mascaro C., Ortega A., Kristie T.M. (2013). A novel selective LSD1/KDM1A inhibitor epigenetically blocks herpes simplex virus lytic replication and reactivation from latency. MBio.

[74] Burgold T., Spreafico F., De Santa F., Totaro M.G., Prosperini E., Natoli G., Testa G. (2008). The histone H3 lysine 27-specific demethylase Jmjd3 is required for neural commitment. PLoS One.

[75] De Santa F., Narang V., Yap Z.H., Tusi B.K., Burgold T., Austenaa L., Bucci G., Caganova M., Notarbartolo S., Casola S. (2009). Jmjd3 contributes to the control of gene expression in LPS-activated macrophages. EMBO J..

[76] Hong S., Cho Y.W., Yu L.R., Yu H., Veenstra T.D., Ge K. (2007). Identification of JmjC domain-containing UTX and JMJD3 as histone H3 lysine 27 demethylases. Proc. Natl. Acad. Sci. USA.

